# Baicalin-geniposide attenuates pulmonary inflammation and vascular injury via HMGB1 blockade: insights from a cerebral ischemia-reperfusion model and implications for pulmonary hypertension

**DOI:** 10.3389/fphar.2026.1822890

**Published:** 2026-04-15

**Authors:** Leying Gao, Qianqian Wu, Xiaoqiu Li, Yu Long, Nan Li

**Affiliations:** 1 School of Modern Chinese Medicine Industry, School of Pharmacy, Chengdu University of Traditional Chinese Medicine, Chengdu, China; 2 NHC Key Laboratory of Nuclear Technology Medical Transformation, Mianyang Central Hospital, School of Medicine, University of Electronic Science and Technology of China, Mianyang, China

**Keywords:** acute lung injury, baicalin, geniposide, HMGB1, inflammation, pulmonary hypertension

## Abstract

**Introduction:**

Pulmonary complications following stroke are a leading cause of death, with no targeted therapies available. Inflammation drives both post‐stroke neurological damage and secondary acute lung injury (CIS-ALI). High mobility group box 1 (HMGB1), a key mediator in cerebral ischemia stroke (CIS), translocates from the nucleus to cytoplasm and is released extracellularly, triggering inflammatory cascades. The combination of two bioactive metabolites, baicalin and geniposide (BG), exhibits anti‐inflammatory and neuroprotective properties, but its efficacy against CIS-ALI remains unexplored. This study investigated BG’s mechanisms using *in vitro* and *in vivo* models.

**Methods:**

This study investigated the mechanisms of BG using *in vitro* and *in vivo* models. *In vitro*, LPS‐stimulated BV2 microglia and RAW264.7 macrophages were co‐cultured. *In vivo*, a rat model of middle cerebral artery occlusion/reperfusion (MCAO/R) was used. BG was administered at doses of 25—50 mg/kg in rats.

**Results:**

BG dose-dependently suppressed pro-inflammatory cytokines (TNF‐α, IL‐1β, IL‐6) and nitric oxide (NO), while attenuating HMGB1 nucleocytoplasmic translocation via JAK2/STAT3 inhibition. BG (25—50 mg/kg) reduced cerebral infarct volume, neurological deficits, and lung edema. Mechanistically, BG blocked HMGB1 nuclear export in ischemic brains, thereby decreasing HMGB1 levels in serum and lungs, and disrupting inflammatory cross-talk.

**Discussion:**

These findings highlight BG’s unique capacity to concurrently mitigate cerebral injury and secondary ALI by targeting the JAK2/STAT3 axis, offering a safe, multi‐targeted strategy against CIS‐related complications. Given the shared pathological features between ALI and PH–including inflammation, vascular hyperpermeability, and JAK2/STAT3‐driven injury cascades–these findings provide a mechanistic rationale for exploring BG as a potential therapeutic candidate for pulmonary hypertension and related pulmonary vascular diseases.

## 1 Introduction

Stroke, a leading cause of global mortality and disability, is frequently complicated by systemic inflammatory responses that drive secondary organ damage (Collaborators, 2024; Feigin et al., 2025). Among these complications, acute lung injury (ALI) emerges as a critical contributor to post-stroke mortality, affecting 7%–38% of patients and exacerbating outcomes through exacerbating systemic inflammation and organ dysfunction (Robba et al., 2020). Despite advances in stroke management, targeted therapies to mitigate cerebral ischemia-reperfusion (CIS)-induced ALI remain elusive. Conventional approaches, such as broad-spectrum antibiotics or immunosuppressants, are limited by drug resistance, off-target effects, and their inability to address the neuroinflammatory cascades that propagate systemic damage (Kishore et al., 2019; Ciubotaru et al., 2025; Tschoe et al., 2020). This unmet clinical demand underscores the urgency to explore novel interventions that concurrently target cerebral injury and its systemic repercussions.

Pulmonary hypertension (PH), a progressive disorder characterized by increased pulmonary vascular resistance, shares key pathological features with ALI, including endothelial dysfunction, inflammatory cell infiltration, and vascular remodeling (Xu et al., 2023b; Na et al., 2024; Yeh et al., 2023). Emerging evidence implicates HMGB1 as a critical mediator in PH pathogenesis, where it promotes pulmonary arterial smooth muscle cell proliferation and vascular remodeling through TLR4/NF-κB and JAK2/STAT3 signaling (Zhang et al., 2023; Wei et al., 2024). Therefore, natural compounds that effectively inhibit HMGB1 nucleocytoplasmic translocation may hold therapeutic potential for both ALI and PH.

Encephalopathy can affect lung health through a variety of neurological, endocrine, and immune pathways (Li et al., 2023). Emerging evidence highlights the pivotal role of the “brain-lung axis” in post-CIS pathophysiology. Studies reveal that post-CIS pathological alterations in distal lung tissue are triggered through inflammatory factor dissemination, neural dysregulation, and immune suppression, with the trans-organ transmission of inflammatory cascades constituting the central mechanism in this process (Li et al., 2023; Kunze et al., 2023). CIS triggers the release of damage-associated molecular patterns (DAMPs) (Kunze et al., 2023). Following CIS, inflammatory reactions lead to an increase in the permeability of the blood-brain barrier (BBB), which plays a crucial role in post-CIS neurological damage and consequent acute lung injury (Li et al., 2023; Takata et al., 2021). As a pivotal damage-associated molecular patterns, high mobility group box 1 (HMGB1) released by ischemic neurons migrates to pulmonary tissue via the compromised BBB(Chen et al., 2025; Li et al., 2025).

The HMGB1 translocated to pulmonary tissue induces endothelial cell activation, thereby increasing capillary permeability and exacerbating pulmonary inflammatory responses (Li et al., 2024; Wang et al., 2025). Simultaneously, through activation of Toll-like receptors (TLRs), it drives localized innate immune activation, recruits circulating immune cells, and stimulates cytokine and chemokine production. These synergistic mechanisms provoke acute pulmonary and systemic immune activation (Xie and Tuxiu, 2022; Guo et al., 2024). HMGB1 has been widely recognized as a potential biomarker of disease due to its involvement in inflammation and release in response to cellular stress. Critically, HMGB1’s nucleocytoplasmic shuttling is regulated by the JAK2/STAT3 pathway, a signaling hub implicated in both neuroinflammation and systemic immune activation (Liu et al., 2023). Thus, interventions targeting HMGB1 dynamics and its upstream regulators could offer dual neuroprotective and pulmonary protective effects—a strategy yet to be fully exploited.

Natural compounds, with their multi-target mechanisms and favorable safety profiles (Bharate and Lindsley, 2024; He et al., 2025), present a promising avenue for addressing this dual pathology. Baicalin and geniposide are bioactive metabolites isolated from *Scutellaria baicalensis* Georgi [Lamiaceae; Scutellariae radix] and *Gardenia jasminoides* Ellis [Rubiaceae; Gardeniae fructus], respectively. These metabolites have demonstrated synergistic anti-inflammatory and neuroprotective effects in preclinical CIS models (Wu et al., 2019; Fangze, 2020; Li et al., 2021). Preclinical studies demonstrate that the combination of baicalin and geniposide (BG) reduces infarct volume and neuroinflammation in rodent CIS models (Wu et al., 2019). However, existing studies have predominantly focused on their isolated neuroprotective roles, leaving their combined efficacy against CIS-induced systemic complications, particularly ALI, unexplored.

In this study, by using *in vitro* BV2 microglia-RAW264.7 macrophage conditioned medium model and *in vivo* middle cerebral artery occlusion/reperfusion (MCAO/R) rats, we systematically evaluated BG’s effects on neuroinflammation, pulmonary injury, and HMGB1 dynamics. Our findings reveal that BG not only reduces cerebral infarct volume and neurological deficits but also attenuates lung edema and cytokine storms. Mechanistically, BG suppresses JAK2/STAT3 activation, preventing HMGB1 nuclear export and subsequent systemic inflammation (Liu et al., 2023; Xu et al., 2023a). These results position BG as a first-in-class dual-action therapy for CIS-related complications, which provides a potential molecular mechanism and drug intervention strategy reference for the treatment of CIS-ALI.

## 2 Materials and methods

### 2.1 Reagents and materials

Baicalin (purity ≥98%) was purchased from Nanjing Zelang Biotechnology Co., Ltd. Geniposide (purity ≥98%) was purchased from Chengdu Must Biotechnology Co., Ltd. CCK8 was purchased from Bioground. Fetal Bovine Serum (FBS) was purchased from Every Green (China). Dulbecco’s modified Eagle’s medium (DMEM) was purchased from Gibco (BRL Co., Ltd., USA). The enzyme-linked immunosorbent assay (ELISA) kit was purchased from ElabScience (Elabscience Biotechnology Co., Ltd.). The NO kit was purchased from Beyotime Biotechnology (China). The Nucleus Extract Kit was purchased from Solarbio (Beijing Solarbio Science and Technology Co., Ltd.).

### 2.2 Cell culture and drug treatment

RAW264.7 and BV2 cell lines were purchased from the Chinese Academy of Sciences cell bank. BV2 cells were cultured in DMEM supplemented with 10% fetal bovine serum and 1% streptomycin/penicillin in a humidified atmosphere containing 5% CO2 at 37 °C. When the concentration of BV2 cells reached 60%, the cultured BV2 cells were inoculated in 6-well plates and randomly divided into the following four groups: control group, model group, BG low dose group and BG high dose group. The BG low dose and the BG high dose were acted for 4 h, and then the cells were stimulated by the addition of LPS (1 μg/mL) in addition to the control group for 24 h. Cell supernatants were collected for pro-inflammatory factor concentrations. The medium was replaced and the supernatant of each group of cells was collected after 24 h as BV2 conditioned media for the culture of RAW264.7 cells, and the cells were collected for Western blot assay.

**FIGURE 1:**
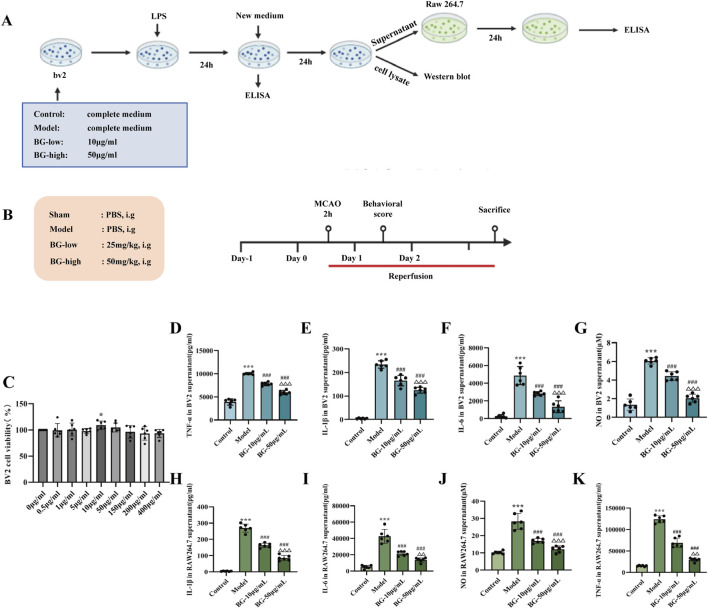
The effect of the BG on the BV2-RAW264.7 cells inflammation models. **(A)** Diagram of the experimental procedure *in vitro*. **(B)** Diagram of the experimental procedure *in vivo*. **(C)** The effect of BG on the viability of BV2 Cells (n = 6). **(D–G)** Expression of TNF-α, IL-1β, IL-6 and NO in BV2 supernatant (n = 6). **(H–K)** Expression of TNF-α, IL-1β, IL-6 and NO in RAW264.7 supernatant (n = 6). Compared with the control group, ^***^ *p* < 0.001; compared with the model group, ^###^ *p* < 0.001; compared with the BG-10 μg/mL group, ^△△^ *p* < 0.01, ^△△△^ *p* < 0.001.

RAW264.7 cells were cultured under the same conditions as BV2 cells, and the cultured RAW264.7 cells were inoculated in 6-well plates and randomly divided into the following four groups: control group, model group, BG low-dose group and BG high-dose group. After 24 h, the collected BV2 CM was used as the medium for treatment. BV2 from each group were collected for Western blot assay and the supernatant was collected after centrifugation of the culture medium. The experimental procedure for the *in vitro* experiments is shown in Figure 1A.

### 2.3 Cell viability assay

The viability of BV2 cells was assessed using the Cell Counting Kit-8 (CCK-8) assay with varying concentrations of BG (Baicalin: Geniposide = 7:3 (Li et al., 2021), 0–400 μg/mL). BV2 cells were seeded with RAW264.7 cells when reaching 60% confluency at 1 × 10^4^ cells per well. Cells were treated with different doses of BG for 24 h, followed by incubation with CCK-8 reagent for 1 h. Absorbance at 450 nm was read on an microplate reader (BIO-RAD, USA).

### 2.4 Measurement of inflammatory cytokines in BV2 and RAW264.7 cells

In order to obtain the modulatory effects of drugs on inflammatory factors in BV2 cells and RAW264.7 cells, the concentrations of TNF-α, IL-1β and IL-6 in the supernatant during the culture of BV2 cells and RAW264.7 cells were determined using an ELISA kit. The absorbance was then measured at 450 nm using an microplate reader (BIO-RAD, USA). In addition, NO levels in the cell culture supernatants of each group were determined using a NO assay kit (Beyotime Biotech, Shanghai, Cat#S0021S, China).

### 2.5 Animals and treatments

Adult male Sprague-Dawley (SD) rats, weighing 270 ± 10 g, were purchased from SPF (Beijing) Biotechnology Co., Ltd. All experimental protocols were approved by the Animal Research Ethics Committee of Chengdu University of Traditional Chinese Medicine (Approval No. 2022–32). Animal data reporting follows ARRIVE 2.0 guidelines.

After all animals were acclimatized for 1 week, rats were randomly divided into four groups: sham group, model (MCAO/R) group, BG- 25 mg/kg group (MCAO/R + BG 25 mg/kg) and BG- 50 mg/kg group (MCAO/R + BG 50 mg/kg). Medication group rats were injected 25 mg/kg or 50 mg/kg BG through the tail vein 1 day before the surgery and daily for 5 consecutive days. The sham and model groups received the same amount of PBS7.0.

### 2.6 Drug configuration

Low-dose BA-GE formulation: Based on a Baicalin: Geniposide ratio of 7:3 (Li et al., 2021), accurately weigh the required amounts of Baicalin and Geniposide, and dissolve them in a predetermined volume of PBS 7.0 to yield a final drug concentration of 5 mg/mL. The solution is then subjected to ultrasonication at ambient temperature for 10 min to ensure uniform dispersion and compensate for any potential volatility. The prepared formulation is temporarily stored at 4 °C in a refrigerator. High-dose BA-GE preparation: Following the same procedure as above, adjust the concentrations to achieve a final drug concentration of 10 mg/mL.

### 2.7 MCAO/R model

The MCAO/R model was performed following the modified Longa method. Briefly, rats were anaesthetized and the left common carotid artery (CCA) and left external carotid artery (ECA) were used for the MCAO/R model. Briefly, rats were anaesthetized and the left CCA and left ECA were isolated and ligated. Clip the internal carotid artery (ICA) and make a small incision, then insert a nylon suture (Beijing cinontech Co., Ltd., China) with round tips with 18–20 mm depth. The wound was then sutured and sterilized. After 2 h of ischaemia, the nylon suture was withdrawn from the ECA back to the CCA to create reperfusion (Belayev et al., 1996). The sham group receive the same postoperative uninserted nylon suture. To ensure the occlusion of MCA and reperfusion, the cortical blood flow was monitored using laser speckle contrast imaging (Liu et al., 2023) (RWD life science, Shenzhen, China). A heating pad was used to maintain the body temperature at 37 °C ± 0.5 °C of rat throughout the surgery. After surgery, animals were initially examined for neurological deficits (double-blind) when fully awake according to Longa 5-point scale (Kapadia et al., 2006), as follows: 0, no deficit; 1, failure to extend contralateral forepaw; 2, spin longitudinally; 3, falling to the right side; 4, unable to walk spontaneously. A score of 1–3 points was the criterion for successful modelling, otherwise the corresponding number of rats was eliminated and supplemented. After 3 days of reperfusion, the rats were sacrificed. The specific procedure is shown in Figure 1B. Postoperatively, iodine-based disinfectant solutions were applied for wound site decontamination. Throughout the postoperative period, animal bedding was replaced daily to maintain aseptic conditions.

### 2.8 Neurological disorder assessment

Neurological function was evaluated at 72 h post-MCAO/R utilizing the Longa 5-point scoring system in a double-blind manner (Kapadia et al., 2006).

### 2.9 Brain infarct size assessment

Rats were anaesthetised and executed by cervical dislocation after which the brains were rapidly removed, placed in a refrigerator at −20 °C for 20 min before being taken out and cut into 2-mm brain sections on ice. The sections were stained with 2% 2,3,5-triphenyltetrazolium chloride (Chengdu kelong chemical Co., Ltd., China) solution for 30 min at 37 °C and fixed in 4% paraformaldehyde. After obtaining photographs of the sections, the infarcted areas were quantitatively analysed by ImageJ software. In addition, cerebral oedema may have a great influence on the volume calculation of cerebral infarction, and the infarct rate was calculated using the following formula: The volumes of the infarctions were calculated using the following formula: corrected infarct volume (%) = [contralateral hemisphere volume − (ipsilateral hemisphere volume-infarct volume)]/contralateral hemisphere volume × 00% (Liu et al., 2023).

### 2.10 Lung wet weight/dry weight ratio (W/D ratio)

The lung W/D ratio is a measure of pulmonary oedema. A portion of the lower lobe of the left lung were excised and the wet weight was recorded. The lung tissue were then placed in an oven at 80 °C until constant weight, and the dry weight recorded. W/D ratio = wet weight/dry weight (Xu et al., 2023a).

### 2.11 Histopathologic evaluation of the brain and lung tissue

We used hematoxylin and eosin (HE) staining to evaluate inflammation in brain and lung tissue. After the animals had been euthanized humanely, transcardial perfusion with 4% paraformaldehyde was performed to fix the tissues. Following this perfusion process, the brain and lung tissues were carefully harvested. Then, the tissues were embedded in paraffin, cut into 5 μm sections, and stained with HE. Finally, the pathological changes of tissues were observed with an optical microscope. Employing the NanoZoomer S60 high-resolution digital scanner, whole-slide imaging was conducted on the histological sections of lung tissue stained with HE.

### 2.12 Semiquantitative histologic grading criteria for lung injury score

Image acquisition was performed on the scanned files using the NDP.view 2 software. Each slide was initially examined at low magnification to survey the entire tissue, followed by capturing micrographs at 12.5-fold and 400-fold magnifications to investigate specific pathologic alterations in detail. All procedures were conducted in a double-blind manner. Lung injury was evaluated by two independent investigators who were blinded to group allocation, using a modified semi-quantitative scoring system (Skydsgaard, 2016). Five histological parameters—alveolar epithelial cell necrosis, alveolar epithelial hyperplasia, inflammatory cell infiltration, hemorrhage, and interstitial edema—were each scored on a scale from 0 to 3, where 0 indicated normal, 1 mild, 2 moderate, and 3 severe. The total lung injury score was calculated as the sum of the individual parameter scores. Inter-rater agreement was high.

### 2.13 Measurement of pro-inflammatory factors in brains and lungs

After homogenisation of brain and lung tissues separately, the ELISA analysis was performed for the detection of inflammatory factors of TNF-α, IL-1β and IL-6 according to the manufacturer’s instructions (Elabscience, Wuhan, China), followed by absorbance measurement at 450 nm using an enzyme labeling instrument (BIO-RAD, USA). To determine NO in brain and lung tissues, Griess assay determination was used to determine the NO levels using the NO determination kit (Shanghai Beyotime Biotech, Cat#S0021S, China) followed by absorbance measurement at 540 nm using an enzyme labeling instrument (BIO-RAD, USA).

### 2.14 Measurement of HMGB1 in brains, lungs and serum

Blood samples were collected from the abdominal aorta at 72 h after reperfusion. Serum was isolated from the blood after centrifugation at 1,500 r/min for 15 min and was frozen at −80 °C until ELISA analyses were performed. HMGB1 concentrations in brain, lung tissues and serum samples were quantified using specific ELISA kits for rats according to the manufacturers’ instructions (Elabscience Biotechnology Co., Ltd.).

### 2.15 Immunofluorescence detection of nucleoplasmic transfer of HMGB1

Fixed BV2 cells and frozen sections of brain tissues were stained using immunofluorescence. BV2 cells and brain tissues were obtained and fixed with 4% paraformaldehyde, closed with 5% BSA, and then incubated with primary antibody HMGB1 (Servicebio, 1:500); they were then incubated with the secondary antibody, and cell nuclei were stained with DAPI and images were taken using a fluorescence microscope (Olympus).

### 2.16 Western blot

We collected BV2 cells and rat brain tissue from each group. Total protein from cultured cells was extracted using RIPA lysis buffer with a protease inhibitor cocktail on ice. Nucleus and cytoplasm proteins were extracted with the Nucleus Extract Kit. Protein concentration was determined for Western blot analysis. Homogenates (50 μg total protein) were electrophoresed on 10% SDS-polyacrylamide gels, transferred to PVDF membranes, and blocked with 5% BSA for 2 h at room temperature. After overnight incubation with primary antibodies at 4 °C, membranes were incubated with secondary antibodies for 1 h at room temperature and stained with enhanced chemiluminescence (ECL) reagent. Target protein levels were imaged and analyzed using a UVP gel analysis system (e-Blot TOUCH IMAGER). Relative protein levels were calculated as densitometric ratios to LaminB1, β-tubulin, or β-actin. Primary antibodies added: anti-JAK2 antibody (CellSignalingTechnology, 1:1,000), anti-STAT3 (Servicebio, 1:500), anti-P-JAK2 (CellSignalingTechnology, 1:1,000), anti-P-STAT3(Servicebio, 1:500), anti-HMGB1 (Servicebio, 1:1,000),anti-β-actin (Servicebio, 1:500),anti-β-tubulin (Proteintech, 1:10,000),anti-LaminB1 (immunoway, 1:1,000). And the relative expression level of each group of proteins was analyzed by ImageJ software (as the ratio of the bands of the target proteins to the bands of the internal control proteins).

### 2.17 Statistical analysis

The data of each group were expressed as mean ± standard deviation. Data significance analysis was performed using GraphPad Prism 9.5.0 (GraphPad Software, San Diego, CA, USA). Statistical comparisons were made using the t-test. Data were considered statistically significant when P < 0.05.

## 3 Result

### 3.1 BG mitigates the release of pro-inflammatory factors from inflammatory microglia and CM treated RAW 264.7 cells

In this study, we simulated the *in vivo* brain-pulmonary interstitial inflammatory cascade response by an *in vitro* BV2-RAW 264.7 cells inflammation model. Figure 1C shows the effect of different doses of BG on the proliferation of BV2 cells. Different doses of 0, 0.5, 1, 5, 10, 50, 150, 200, and 400 μg/mL of BG solution were applied to normal BV2 cells for 2 h. Compared with the normal group of cells, the intervention of different doses of BG solution had almost no inhibitory effect on the proliferation of BV2 cells. Therefore, BG solution is safe for pharmacologic intervention in the dose range of 0–400 μg/mL. In this study, 10, 50 μg/mL were chosen as the low and high dose groups of BG solution for the study. As shown in Figures 1D–G, in BV2 cells, the levels of pro-inflammatory factors TNF-α, IL-1β, IL-6 and NO secreted by LPS-treated BV2 cells were significantly elevated compared with those of the normal group, suggesting that LPS induced the release of pro-inflammatory factors from BV2 cells by generating inflammatory responses. Compared with the model group, the levels of TNF-α, IL-1β, IL-6 and NO secreted by the cells in the BG low and high dose groups were significantly lower (P < 0.01) and showed a dose-dependent pattern. As shown in Figures 1H–K, in RAW 264.7 cells, CM from BV2 resulted in significantly higher levels of TNF-α, IL-1β, IL-6 and NO in RAW 264.7 cells compared with normal medium (P < 0.001), indicating that *in vitro* model of the inflammatory crosstalk between BV2 cells and RAW264.7 cells was successfully established. The levels of pro-inflammatory factors TNF-α, IL-1β, IL-6, and NO released by RAW264.7 cells were significantly reduced after the intervention of low and high doses of BG, and the intervention effect was better in the high dose group (P < 0.5, P < 0.001, P < 0.5, P < 0.5). According to the above results, it can be found that the low and high doses of BG had no effect on the levels of normal cellular inflammatory factors, and could reduce the expression of pro-inflammatory factors in the inflammatory cascade response between BV2-RAW264.7.

### 3.2 BG modulates the JAK2-STAT3 pathway in the LPS-induced BV2-RAW264.7 *in vitro* inflammation model

**FIGURE 2:**
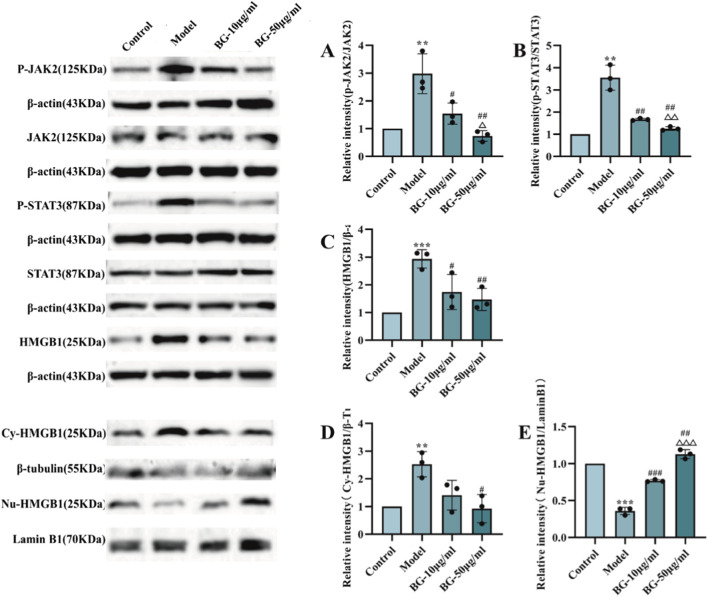
Effect of BG on JAK2/STAT3/HMGB1 signaling pathway in LPS-induced BV2. **(A)** Quantification of the p-JAK2/JAK2 in LPS-induced BV2 (n = 3). **(B)** Quantification of the p-STAT3/STAT3 in LPS-induced BV2 (n = 3). **(C)** Quantification of the HMGB1 in LPS-induced BV2 (n = 3). **(D)** Quantification of the HMGB1 in the cytoplasm in LPS-induced BV2 (n = 3). **(E)** Quantification of the HMGB1 in the nucleus in LPS-induced BV2 (n = 3). Compared with the control group, ^**^ *p* < 0.01, ^***^ *p* < 0.001; compared with the model group, ^#^ *p* < 0.05, ^##^ *p* < 0.01, ^###^ *p* < 0.001; compared with the BG-10 μg/mL group, ^△^ *p* < 0.05, ^△△^ *p* < 0.01, ^△△△^ *p* < 0.001.

LPS activates JAK2 in cells *in vitro* to further produce inflammatory factors involved in the initiation of the immune cascade. As shown in Figures 2A–C, Western blot results showed that p-JAK2/JAK2, p-STAT3/STAT3 and HMGB1 in BV2 cells were significantly increased by LPS (P < 0.05) and significantly decreased by BG (P < 0.05) in a dose-dependent manner. The results suggest that BG may reduce the production of inflammatory factors by inhibiting p-JAK2/JAK2, p-STAT3/STAT3, and HMGB1. Meanwhile, the results of Western blot after nucleoplasmic protein separation were shown in Figures 2D,E, compared with the control group, HMGB1 in the cytoplasm was significantly increased after 72 h of CIS, while HMGB1 in the nucleus was significantly reduced (P < 0.05), suggesting that BG may reduce the nucleoplasmic translocation of HMGB1 by inhibiting JAK2-STAT3 pathway.

### 3.3 BG ameliorates neurological injury in MCAO/R rats

#### 3.3.1 BG reduces infarct volume and improves neurologic deficits in rats 3 days after MCAO/R

**FIGURE 3:**
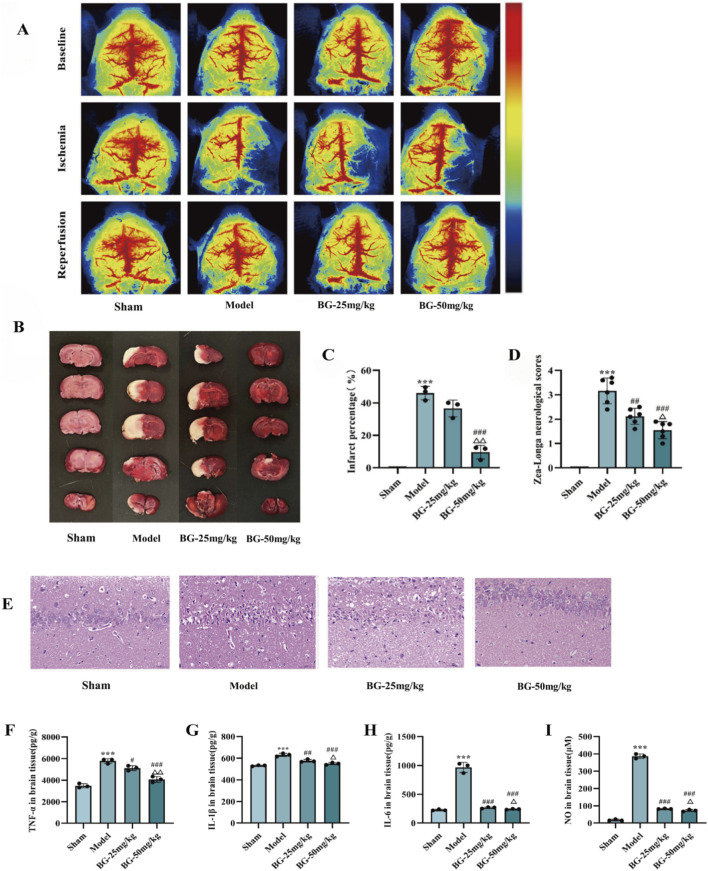
Effect of BG on brain damage in MCAO/R rats. **(A)** Cerebral blood flow monitored using 2-dimensional laser speckle imaging techniques before, during MCAO, and reperfusion. **(B)** Representative brain slices were stained with TTC. **(C)** Infarct volume quantitative analysis (n = 3). **(D)** Zea-Longa neurological scores (n = 6). **(E)** HE staining of brain tissue. **(F–I)** Expression of TNF-α, IL-1β, IL-6 and NO in brain tissues (n = 3). Compared with the sham group, ^***^ *p* < 0.001; compared with the model group, ^#^ *p* < 0.05, ^##^ *p* < 0.01, ^###^ *p* < 0.001; compared with the BG-25 mg/kg group, ^△^ *p* < 0.05, ^△△^ *p* < 0.01.

The neuroprotective effect of BG was evaluated after the MCAO/R model was constructed. To objectively confirm the success of ischemia and reperfusion, cortical cerebral blood flow (CBF) was monitored in real-time using laser speckle contrast imaging (LSCI). As shown in Figure 3A, CBF in the ipsilateral hemisphere dropped sharply (to less than 30% of baseline) immediately upon suture insertion, indicating successful middle cerebral artery occlusion. Upon suture withdrawal, CBF rapidly recovered to over 70% of baseline levels, confirming effective reperfusion. TTC staining showed that administration of BG (50 mg/kg) significantly reduced infarct volume compared with the model group (P < 0.001) (Figures 3B,C). Figure 3D showed that BG significantly attenuated neurological deficits compared with the model group (P < 0.01, P < 0.001). In addition, compared with the BG low-dose group, the BG high dose had a greater protective effect, showing a dose-dependence.

#### 3.3.2 BG improves the histopathologic evaluation of brain tissues

The brain tissues of rats in each group were processed by HE staining to assess the modulating effect of the drug on the brain histopathology of model rats. As shown in Figure 3E, the nerve cells of rats in the sham group were neatly arranged and regular in morphology, with no obvious damage. In the model group, the neuronal cells were edematous and showed large necrotic changes, the nuclei of the cells were crumpled and deeply stained, and the cells could be seen as vacuolated changes. After the administration of BG (25 and 50 mg/kg), the pathological changes of rat nerve cells were significantly reduced compared with that of the model group, and the cells were relatively neatly arranged, the cytoplasm was fuller and richer than that of the model group, and the vacuolated changes were significantly reduced. At the same time, the improvement of cell nuclei crumpling and cell vacuolization was better in the BG high dose group than in the BG low dose group.

#### 3.3.3 BG reduces pro-inflammatory factors of brains

Inflammatory markers TNF-α (Figure 3F), IL-1β (Figure 3G), IL-6 (Figure 3H) and NO (Figure 3I) levels in brain tissues were measured to assess inflammation. Compared to the normal group, the model group exhibited significantly increased TNF-α, IL-1β, IL-6, and NO levels (P < 0.001, P < 0.01, P < 0.01, P < 0.01, P < 0.001), indicating brain inflammation post-MCAO. BG intervention notably reduced these pro-inflammatory factors, with the high-dose group showing superior anti-inflammatory effects compared to the low-dose group.

### 3.4 BG ameliorates lung injury in MCAO/R rats

#### 3.4.1 BG ameliorates pulmonary edema

**FIGURE 4:**
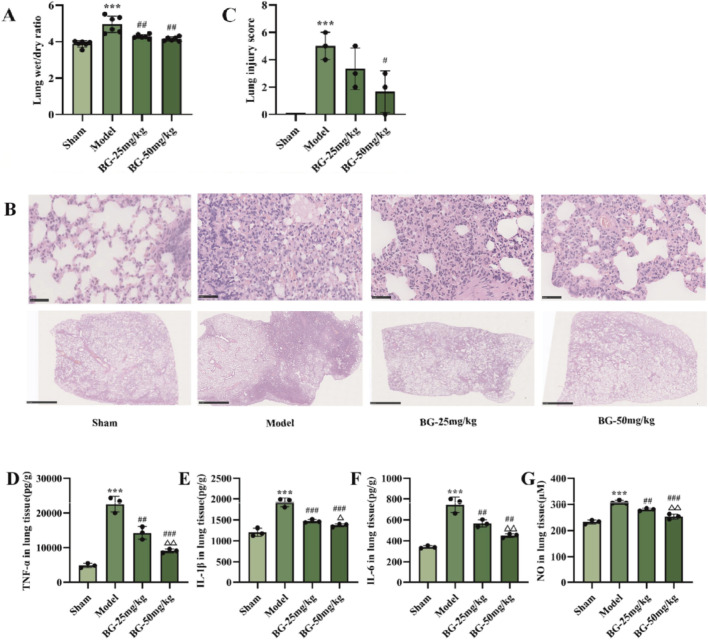
Effect of BG on lung injury in MCAO model rats. **(A)** Lung W/D ratio (n = 6). **(B)** HE staining representative pictures of lung tissue. In the model group, alveolar epithelial necrosis, alveolar epithelial hyperplasia, inflammatory cell infiltration, alveolar septal thickening, hemorrhage and cellulose exudation were observed. The above pathological changes were alleviated in BG treatment group. Ruler = 50 μm. **(C)** Lung injury score (n = 3). **(D–G)** Expression of TNF-α, IL-1β, IL-6 and NO in lung tissues (n = 3). Compared with the sham group, ^***^ *p* < 0.001; compared with the model group, ^#^ *p* < 0.05, ^##^ *p* < 0.01, ^###^ *p* < 0.001; compared with the BG-25 mg/kg group, ^△^ *p* < 0.05, ^△△^ *p* < 0.01.

Acute lung injury from CIS leads to pulmonary edema and alveolar enlargement. Post-sacrifice, the impact of BG on CIS-induced pulmonary edema was evaluated by measuring the W/D ratio of lungs. Figure 4A shows a significant increase lung W/D ratio in the model group versus the sham group. However, lung tissue W/D ratio significantly reduced (P < 0.05) with BG treatment at doses of 25 and 50 mg/kg.

#### 3.4.2 BG ameliorates the histopathologic evaluation of lung tissues

As shown in Figure 4B, in the model group, histopathological changes in the lung tissue were observed, including degeneration and necrosis of alveolar epithelial cells, proliferation of alveolar epithelial cells, infiltration of inflammatory cells, fibrous tissue hyperplasia, fibrin-like exudation, and hemorrhage. After administration of BG, these pathological alterations showed some degree of alleviation. As shown in Figure 4C, the statistical results indicate a significant increase in lung tissue injury scores in the model group compared to the sham group (P < 0.01). In comparison with the model group, the BG-50 mg/kg group exhibited a noticeable decrease in lung tissue injury scores (P < 0.05). On the other hand, the BG-25 mg/kg group demonstrated a certain decrease in injury scores, but this difference was not statistically significant (P > 0.05).

#### 3.4.3 BG reduces pro-inflammatory factors in the lungs

In order to verify the effect of the drugs on the inflammatory cascade response between the hindbrain and lungs in the MCAO model, relevant pro-inflammatory factors were examined in the lung tissues of the rats in each group. As shown in Figure 4, compared with the sham group, the levels of TNF-α (Figure 4D), IL-1β (Figure 4E), IL-6 (Figure 4F) and NO (Figure 4G) in the lung tissues of the model group were significantly increased, suggesting that cerebral ischemia causes inflammation in the lungs and triggers lung injury. In contrast, BG treatment had a significant inhibitory effect on pro-inflammatory factors in the lungs of the MCAO rat model, and showed a dose-related effect.

### 3.5 BG modulates the JAK2-STAT3 pathway in rat brain tissue of the MCAO model to reduce nucleoplasmic translocation of HMGB1

**FIGURE 5:**
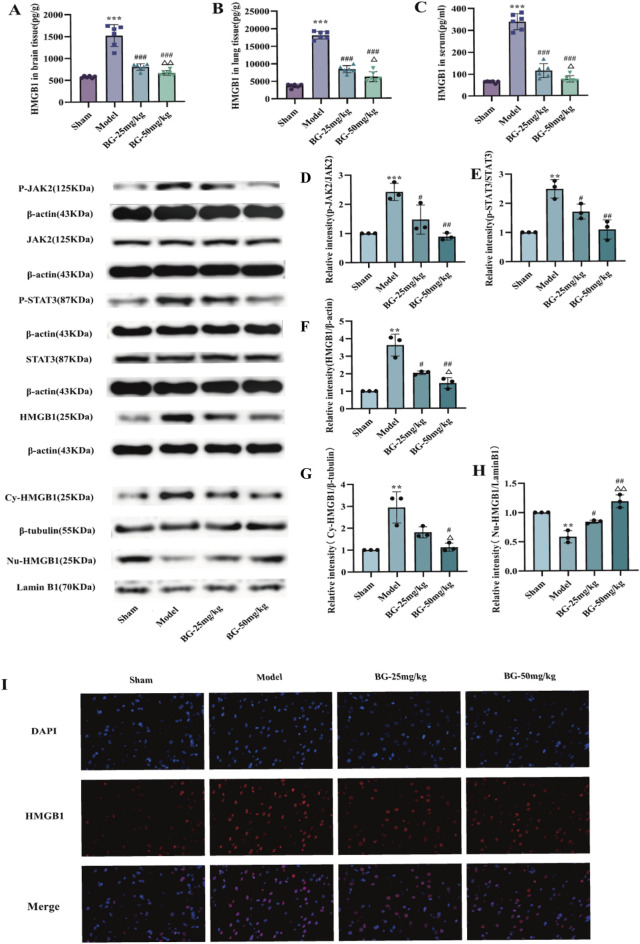
Effect of BG on JAK2/STAT3/HMGB1 signalling pathway in brain tissue of MCAO rats. **(A–C)** Expression of HMGB1 in brain, lung tissues and serum (n = 6). **(D)** Quantification of the p-JAK2/JAK2 in brains (n = 3). **(E)** Quantification of the p-STAT3/STAT3 in brains (n = 3). **(F)** Quantification of the HMGB1 in brains (n = 3). **(G)** Quantification of the HMGB1 in the cytoplasm in brains (n = 3). **(H)** Quantification of the HMGB1 in the nucleus in brains (n = 3). **(I)** Immunofluorescence assay of HMGB1 in brain tissues. Compared with the sham group, ^**^ *p* < 0.01, ^***^ *p* < 0.001; compared with the model group, ^#^ *p* < 0.05, ^##^ *p* < 0.01, ^###^ *p* < 0.001; compared with the BG-25 mg/kg group, ^△^ *p* < 0.05, ^△△^ *p* < 0.01.

The MCAO/R model triggers an inflammatory cascade response, which further produces inflammatory factors involved in the initiation of the immune cascade response. ELISA results as shown in Figures 5A–C that HMGB1 levels in brain, lung tissues and serum were significantly elevated in the model group (P < 0.001), and the above responses were reversed after administration of BG (P < 0.001), indicating that the drug intervention reduced the levels of HMGB1 in the brain, lung tissues as well as in the serum of the MCAO/R rats. Western blot results as in Figures 5D–F showed that the expression levels of p-JAK2/JAK2, p-STAT3/STAT3, and HMGB1 in rats of the model group were significantly increased after 72 h of CIS (P < 0.05). The expression levels of the above proteins were reversed after BG administration, and the drug intervention significantly reduced the expression of p-JAK2/JAK2, STAT3/p-STAT3, and HMGB1 in a dose-dependent manner, suggesting that low and high doses of BG may reduce inflammatory responses by inhibiting the expression of the above proteins. The results of Western blot after nucleoplasmic protein separation were shown in Figures 5G,H, the HMGBI protein in the sham group was mainly located in the nucleus, and the cytoplasm contained only a small amount of HMGB1. Compared with the sham group, the HMGB1 in the cytoplasm was significantly elevated after 72 h of CIS, while the HMGB1 in the nucleus was significantly reduced (P < 0.05), suggesting that BG may reduce the nucleoplasmic translocation of HMGB1 by inhibiting the JAK2-STAT3 pathway.

The existence of HMGB1 as a damage associated molecular patterns that activates inflammatory signaling pathways presupposes its translocation from the nucleus to the cytoplasm followed by its release into the extracellular space. To investigate the effect of BG on the nuclear translocation of HMGBI after MCAO, the nucleoplasmic translocation of HMGB1 in the brain tissues of rats in each group was explored by immunofluorescence. As shown in Figure 5I, HMGB1 in the sham group was mainly located in the nucleus with no cytoplasmic staining. In contrast, fluorescent staining of HMGB1 in the periphery of the nucleus was seen in the model group, suggesting translocation of intracellular HMGB1 from the nucleus to the cytoplasm in the midbrain after CIS, suggesting that intracellular HMGB1 translocated from the nucleus to the cytoplasm in the brain after CIS. Meanwhile, the extracellular nuclear HMGB1 was reduced after the intervention of BG group (low and high dose), suggesting that the intervention of BG could reduce the nucleoplasmic translocation of HMGB1 in the brain of MCAO/R model.

### 3.6 BG reduces nucleoplasmic translocation of HMGB1 by blocking the JAK2/STAT3 signalling pathway in BV2 cells

**FIGURE 6:**
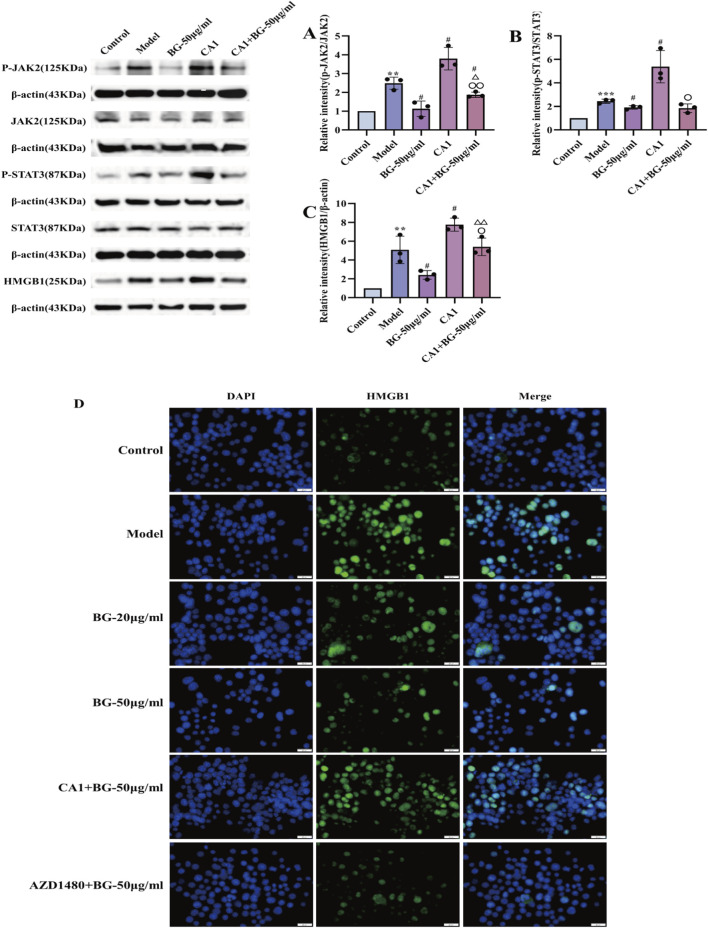
In LPS-induced BV2, BG reduces JAK2 activation and blocks the JAK2-STAT3-HMGB1 pathway, thereby reducing the inflammatory response. **(A)** Quantification of the p-JAK2/JAK2 in BV2 (n = 3). **(B)** Quantification of the p-STAT3/STAT3 in BV2 (n = 3). **(C)** Quantification of the HMGB1 in BV2 (n = 3). **(D)** Immunofluorescence assay of HMGB1. Compared with the control group, ^**^ *p* < 0.01, ^***^ *p* < 0.001; compared with the model group, ^#^p < 0.05; compared with the BG-50 μg/mL group, ^△^ *p* < 0.05, ^△△^ *p* < 0.01; compared with the CA1 group, ^○^ *p* < 0.05, ^○○^ *p* < 0.01.

To further investigate the mechanism by which BG regulates nucleoplasmic translocation of HMGB1, BV2 cells were pretreated with coumermycin A1 (CA1, JAK2 activator, 10 μM) prior to LPS stimulation, and then incubated with BG (50 μg/mL) for 24 h. Western blot assay during CA1 pretreatment showed a significant increase in the expression of p-JAK2, p-STAT3 and HMGB1 expression was significantly increased, and the effect of coumermycin A1 was partially eliminated by BG treatment compared with CA1 treatment (Figures 6A–C). These results suggest that BG partially blocked the HMGB1-induced inflammatory response by inhibiting the JAK2/STAT3 pathway.

In addition, we added AZD1480 (JAK2 inhibitor, 1 μM) group when performing immunofluorescence assay, AZD1480 pretreated BV2 and then incubated with BG (50 μg/mL) for 24 h. The results were shown in Figure 6D, the immunofluorescence results showed that LPS treatment enhanced the green fluorescence outside of the nucleus, indicating that LPS significantly upregulated the intracellular HMGB1 translocation from the nucleus to cytoplasmic translocation of BV2 cells. The green fluorescence outside the nucleus was weakened by BG administration treatment, indicating that it inhibited the nucleoplasmic translocation of HMGB1. The inhibition of the nucleoplasmic translocation of HMGB1 by BG was partially offset with the addition of JAK2 agonists. The addition of JAK2 inhibitors synergistically decreased the nucleoplasmic translocation of HMGB1 caused by LPS in conjunction with BG. These results suggest that BG is able to prevent the nucleoplasmic translocation of HMGB1 by inhibiting the JAK2/STAT3 pathway, which in turn prevents the inflammatory response induced by HMGB1 release.

## 4 Conclusion

In this study, we successfully established an *ex vivo* and *in vivo* CIS-ALI model by simulating the inflammatory cascade during ALI resulting from post-CIS. The experimental data showed that inflammatory damage in brain and lung tissues was inhibited by modulating inflammatory factors in the inflammatory response after the combination of BA and GE, thus ameliorating lung function after CIS, and this effect was related to the inhibition of HMGB1 nucleoplasmic transfer in the brain by suppressing the JAK2/STAT3 signaling pathway.

## 5 Discussion

Inflammatory responses triggered by CIS are key drivers of secondary brain damage and remote organ injury, such as in the lungs. HMGB1, a DAMP, translocates from the nucleus to the cytoplasm under ischemic conditions and enters the circulatory system via the blood-brain barrier, activating downstream signaling pathways and triggering inflammatory cascades. This study confirms through immunofluorescence and Western blot that BG inhibits HMGB1 nucleocytoplasmic shuttling, reducing its release as a pro-inflammatory mediator. Notably, HMGB1 translocation depends on JAK2/STAT3 pathway activation. JAK2, a member of the protein tyrosine kinase family, phosphorylates (p-JAK2) under ischemic conditions, promoting STAT3 nuclear translocation. Research shows that nuclear translocation of p-STAT3 induces HMGB1 transcription and triggers inflammatory responses (Deng et al., 2020; Liu et al., 2023).

BG has a wide range of clinical applications as a traditional combination. Our study provides compelling evidence that BG can inhibit this nucleocytoplasmic shuttling of HMGB1. By suppressing the phosphorylation of JAK2 and STAT3, BG reduces the transcriptional activation of HMGB1 and prevents its release from ischemic neurons. This is supported by our *in vitro* findings in LPS-stimulated BV2 microglia and *in vivo* observations in MCAO/R rats (Belayev et al., 1996), where BG treatment led to decreased cytoplasmic HMGB1 levels and reduced HMGB1 staining in the extracellular space. The inhibition of HMGB1 release is particularly significant as it disrupts the propagation of inflammatory signals from the brain to the lungs (Samary et al., 2018; Kalsotra et al., 2007; Mascia, 2009), a mechanism that is gaining traction in the field of neuroinflammation research.

While Baicalin and Geniposide have been discussed as potential pan-assay interference compounds (PAINS) in certain biochemical screening contexts (Magalhães et al., 2021; Bolz et al., 2021), the core findings of our study are substantiated by robust *in vivo* evidence. The observed therapeutic effects—including significant reductions in cerebral infarct volume, neurological deficits, pulmonary edema, and serum HMGB1 levels in the MCAO/R rat model—cannot be explained by *in vitro* assay artifacts. These *in vivo* pharmacological activities, along with the dose-dependent inhibition of the JAK2/STAT3 pathway, strongly support the biological relevance and target specificity of the baicalin–geniposide combination in the context of CIS-ALI.

The concept of the brain-lung axis has emerged as a critical framework for understanding the systemic consequences of CIS (Xie and Tuxiu, 2022; Mascia, 2009; Kunze et al., 2023). Post-CIS the disruption of the BBB allows the escape of DAMPs like HMGB1 into the systemic circulation. These molecules then travel to the lungs, where they induce endothelial cell activation, increase capillary permeability, and exacerbate pulmonary inflammation (Samary et al., 2018; Wu et al., 2006; Veeravagu et al., 2014). This communication between the brain and lungs is not merely a passive consequence of CIS but an active inflammatory pathway that amplifies organ damage.

BG’s ability to mitigate both neurological and pulmonary injury underscores its dual-action mechanism. By targeting the JAK2/STAT3-HMGB1 axis in the brain (Deng et al., 2020; Wu et al., 2019; Li et al., 2021), BG reduces the initial inflammatory insult and subsequent distant organ involvement. Our *in vivo* data demonstrate that BG treatment not only decreases brain infarct volume and improves neurological scores (Belayev et al., 1996; Kapadia et al., 2006) but also attenuates pulmonary edema and histopathological injury in the lungs (Samary et al., 2018; Veeravagu et al., 2014).

While the conditioned medium approach using BV2 and RAW264.7 cells effectively simulates inflammatory crosstalk, it does not capture dynamic cell–cell interactions. Future studies employing transwell or microfluidic co-culture systems could provide additional insights into the direct communication between microglia and macrophages. Additionally, BG was administered prophylactically in this study; future studies should evaluate post-insult therapeutic dosing schedules to better model clinical translation.

The pulmonary pathological features observed in our CIS model—inflammatory infiltration, vascular hyperpermeability, and HMGB1-mediated injury cascades—are hallmark events shared with PH. In PH, persistent inflammation and endothelial dysfunction drive pulmonary vascular remodeling (Pei et al., 2025; Rich et al., 2018). HMGB1 has been increasingly recognized as a key contributor to PH progression, activating pulmonary arterial smooth muscle cell proliferation via JAK2/STAT3 signaling (Chen and He, 2020; Bauer et al., 2012). Our findings demonstrate that BG effectively blocks HMGB1 translocation and attenuates pulmonary inflammation, suggesting potential efficacy in PH. The marked reduction in pulmonary vascular hyperpermeability and inflammation observed in our study strongly supports future investigation of BG in PH-specific models, such as monocrotaline-induced or hypoxia-induced PH rats.

In summary, our study elucidates the molecular mechanisms by which BG exerts its neuroprotective and anti-inflammatory effects, highlighting the JAK2/STAT3-HMGB1 axis as a key target (Deng et al., 2020; Wu et al., 2019; Xu et al., 2023a). By bridging neuroprotection and systemic anti-inflammatory actions, BG presents a novel strategy for managing CIS and its complications. While challenges remain in translating these findings to the clinic, the promising results from our preclinical models warrant further investigation and offer hope for improved outcomes in CIS patients and potentially those with PH.

## Data Availability

The datasets presented in this study can be found in online repositories. The names of the repository/repositories and accession number(s) can be found below: 10.6084/m9.figshare.31487383.
